# Prevalence of motor development delay in children with craniosynostosis: a systematic review and meta-analysis

**DOI:** 10.1007/s00431-026-06980-w

**Published:** 2026-05-01

**Authors:** Vanessa Grymuza de Souza, Mateus Stanoga Santos, Lais Panno, Francyelle dos Santos Soares, Gladson Ricardo Flor Bertolini, Lucineia de Fátima Chasko Ribeiro

**Affiliations:** 1 Centro de Atenção e Pesquisa em Anomalias Craniofaciais, Hospital Universitário do Oeste do Paraná, Universitaria St. 2069, Cascavel, Paraná 85819-110 Brazil; 2 Universidade Estadual do Oeste do Paraná, Cascavel, Paraná Brazil

**Keywords:** Motor skills, Infants, Craniofacial abnormalities

## Abstract

Craniosynostosis (CS) may influence motor development in childhood through anatomical and functional alterations resulting from premature fusion of cranial sutures. Although several studies have addressed neuropsychomotor outcomes in CS, the lack of consolidated estimates of motor delay prevalence represents a relevant scientific gap, which motivated the present study. This systematic review and meta-analysis aimed to estimate the prevalence of motor development delay in children with CS. Seven electronic databases, gray literature sources, and reference lists of included studies were systematically searched. Observational studies reporting quantitative data on motor performance in children with CS, assessed using standardized instruments, were included. Risk of bias was evaluated using the Joanna Briggs Institute (JBI) Checklist for Prevalence Studies. A total of 8 studies published between 2001 and 2024, involving 510 children with CS, were included. The samples were predominantly non-syndromic, although one study included a small syndromic subgroup (*n* = 8). The meta-analysis demonstrated a pooled preoperative prevalence of motor delay of 31% (95% CI: 20–44%) with substantial heterogeneity across studies (I^2^ = 85.8%).

*Conclusion*: Overall, the findings indicate that approximately one in three children with CS presents some degree of motor delay, reinforcing the need for standardized motor assessments, particularly during the preoperative period.

**Table:** 

**What Is Known:** *• Craniosynostosis (CS) is a congenital condition that may impair neuropsychomotor development due to anatomical and functional brain alterations.* *• Previous studies have reported heterogeneous findings on motor outcomes in children with CS, but no systematic review or meta-analysis had consolidated the prevalence of motor delay, particularly in the preoperative period.* **What Is New:** *• This meta-analysis provides the first pooled estimate of motor delay prevalence in children with non‑syndromic CS, showing that approximately one in three children (36%; 95% CI: 28–44%) presents with motor delay before surgery.* *• The findings highlight the high frequency of motor impairment across all suture subtypes and underscore the urgent need for standardized preoperative motor screening and early multidisciplinary intervention. *

**What Is Known:**

*• Craniosynostosis (CS) is a congenital condition that may impair neuropsychomotor development due to anatomical and functional brain alterations.*

*• Previous studies have reported heterogeneous findings on motor outcomes in children with CS, but no systematic review or meta-analysis had consolidated the prevalence of motor delay, particularly in the preoperative period.*

**What Is New:**

*• This meta-analysis provides the first pooled estimate of motor delay prevalence in children with non‑syndromic CS, showing that approximately one in three children (36%; 95% CI: 28–44%) presents with motor delay before surgery.*

*• The findings highlight the high frequency of motor impairment across all suture subtypes and underscore the urgent need for standardized preoperative motor screening and early multidisciplinary intervention.
*

## Introduction

Craniosynostosis (CS) is a congenital craniofacial malformation characterized by the premature fusion of one or more cranial sutures, which restricts normal skull growth in the direction perpendicular to the affected suture [1]. The estimated global prevalence is approximately 5.9 per 10,000 live births, with most cases being non-syndromic (5.2 per 10,000 births) and a smaller proportion associated with genetic syndromes, in which multiple sutures are affected [2, 3]. In non-syndromic forms, fusion typically involves a single suture, most commonly the sagittal suture (55–60%), followed by the coronal (20–25%), metopic (≈15%), and lambdoid sutures (3–5%) [4].

The etiology of CS is considered multifactorial, resulting from the interaction of genetic, environmental, and mechanical factors. Genetic contributors include mutations in genes that promote premature ossification of cranial sutures [5]. Environmental factors include maternal conditions such as diabetes, smoking, antidepressant use during pregnancy, and thyroid dysfunction, whereas mechanical factors include intrauterine constraint [6].

Premature suture fusion may compromise normal skull and brain development, leading to morphological and functional alterations and an increased risk of elevated intracranial pressure (ICP), sensory and cognitive deficits, and the occurrence of hydrocephalus, particularly in syndromic forms [7, 8]. These structural alterations have been proposed as potential mechanisms underlying the motor delay observed in children with CS, as they may involve cortical reorganization, sensorimotor dysfunction, and visuospatial impairments resulting from craniofacial deformities secondary to compensatory skull growth [4, 9].

The progressive acquisition of gross motor skills—those involving the control of large muscle groups—and fine motor skills—requiring greater precision—is essential for environmental exploration, social interaction, and cognitive development [10]. Delays in the acquisition of these skills may indicate neurological impairment and negatively affect child development, as motor development is closely related to cognition, socialization, and physical health, serving as a sensitive indicator of overall neuropsychomotor functioning [11].

Although CS may influence motor performance by reflecting the maturation and integrity of the central nervous system, much of the literature has primarily focused on cognitive outcomes or postoperative assessments [12–17]. Several systematic reviews and meta-analyses have investigated neurodevelopmental outcomes in children with CS across different suture types, including cognitive, behavioral, and psychological functioning [18–21]. However, in these studies, motor development was typically considered only as a secondary outcome rather than a primary focus of analysis. Studies specifically addressing motor performance, particularly in the preoperative period, remain limited and have reported heterogeneous findings regarding the magnitude of delays and their association with specific suture subtypes [26, 27]. Consequently, despite the potential impact of CS on overall development, motor outcomes remain insufficiently explored and poorly consolidated in the literature.

To date, no systematic reviews or meta-analyses have synthesized evidence on the prevalence of motor delay in children with CS. Importantly, evaluating motor development during the preoperative period may provide a clearer understanding of the direct impact of CS on early neurodevelopment, since surgical intervention may alter developmental trajectories and introduce additional heterogeneity across studies. Therefore, the aim of this systematic review and meta-analysis was to estimate the prevalence of motor delay in children with CS and to synthesize available evidence to inform early diagnostic strategies and multidisciplinary intervention.

## Methods

### Study design and registration

This systematic review and meta-analysis was conducted in accordance with the Preferred Reporting Items for Systematic Reviews and Meta-Analyses (PRISMA) checklist [24]. Prior to data extraction, the review protocol was registered in the Open Science Framework (OSF) database (10.17605/OSF.IO/3BYG7).

### Search strategy

A preliminary search was conducted in PubMed, PROSPERO (International Prospective Register of Systematic Reviews), the Cochrane Database of Systematic Reviews, DARE (Database of Abstracts of Reviews of Effects), and JBI Evidence Synthesis (Joanna Briggs Institute) to identify existing or ongoing reviews addressing the same topic. No eligible reviews were identified; therefore, this systematic review was undertaken.

The search strategy combined relevant keywords and free-text terms, including synonyms, to identify studies related to exposure (craniosynostosis) and the outcome (prevalence of motor delay). No restrictions were applied regarding language or publication date to ensure comprehensive coverage of the literature. The final search strategy developed for PubMed (Table 1) was adapted for each database (EMBASE, Scopus, Web of Science, CINAHL, and LILACS) and for gray literature sources (Google Scholar). Reference lists of the included studies were also manually screened.

**Table 1:** Search in the literature

Source	Strategy (16.09.2025)	Hits
1.PubMed	("Motor Skills Disorders"[MeSH Terms] OR "motor skills disord*"[Title] OR "Developmental Coordination Disorder"[Title] OR "Developmental Coordination Disorders"[Title] OR "Developmental Disabilities"[MeSH Terms] OR "developmental disabilit*"[Title] OR "Child Development Disorders"[Title] OR "Developmental Delay Disorders"[Title] OR "neurodevelopment*"[Title]) AND ("Craniosynostoses"[MeSH Terms] OR "craniosynostos*"[Title] OR "craniostenos*"[Title] OR "Synostotic Plagiocephaly"[Title] OR "Acrocephaly"[Title] OR "Oxycephaly"[Title] OR "lambdoid synostos*"[Title] OR "Synostotic Anterior Plagiocephaly"[Title] OR "Unilateral Coronal Synostosis"[Title] OR "Unilateral Coronal Synostosis"[Title] OR "Trigonocephaly"[Title] OR "Scaphocephaly"[Title] OR "Sagittal Synostosis"[Title] OR "Metopic Synostosis"[Title] OR "Metopic Synostoses"[Title] OR "Single Suture"[Title] OR "Cranial Synostosis"[Title])	164
2. Embase	(‘craniosynostosis’/exp OR ‘craniosynostosis’ OR ‘cranial synostosis’/exp OR ‘cranial synostosis’ OR ‘unilateral coronal synostosis’ OR ‘scaphocephaly’ OR ‘trigonocephaly’ OR ‘metopic synostosis’ OR ‘sagital synostosis’ OR ‘lambdoid synostosis’) AND (‘motor skills disorders’/exp OR ‘motor skills disorders’ OR ‘developmental coordination disorders’ OR ‘neurodevelopmental’/exp OR ‘neurodevelopmental’ OR ‘developmental disabilities’ OR ‘developmental delay disorders’)	528
3. Scopus	TITLE-ABS-KEY ("craniosynostosis" OR "cranial synostosis" OR "unilateral coronal synostosis" OR "scaphocephaly" OR "trigonocephaly" OR "metopic synostosis" OR "sagittal synostosis" OR "lambdoid synostosis") AND TITLE-ABS-KEY ("motor skills disorders" OR "developmental coordination disorders" OR "neurodevelopmental" OR "developmental disabilities" OR "developmental delay disorders")	325
4. Web of Science	"craniosynostosis" OR "craniosynostos*" OR "craniostenos*" OR "Synostotic Plagiocephaly" OR "Acrocephaly" OR "Oxycephaly" OR "lambdoid synostos*" OR "Synostotic Anterior Plagiocephaly" OR "Unilateral Coronal Synostosis" OR "Trigonocephaly" OR "Scaphocephaly" OR "Sagittal Synostosis" OR "Metopic Synostosis" OR "Metopic Synostoses" OR "Single Suture" OR "Cranial Synostosis"	92
5.Lilacs	("craniosynostosis" OR "craniosynostos*" OR "craniostenos*" OR "Synostotic Plagiocephaly" OR "Acrocephaly" OR "Oxycephaly" OR "lambdoid synostos*" OR "Synostotic Anterior Plagiocephaly" OR "Unilateral Coronal Synostosis" OR "Trigonocephaly" OR "Scaphocephaly" OR "Sagittal Synostosis" OR "Metopic Synostosis" OR "Metopic Synostoses" OR "Single Suture" OR "Cranial Synostosis")	160
6.CINAHL	TI ("craniosynostosis" OR "cranial synostosis" OR "unilateral coronal synostosis" OR "scaphocephaly" OR "trigonocephaly" OR "metopic synostosis" OR "sagittal synostosis" OR "lambdoid synostosis") AND AB ("motor skills disorders" OR "developmental coordination disorders" OR "neurodevelopmental" OR "developmental disabilities" OR "developmental delay disorders")	114
8. Google Scholar	(craniosynostosis OR cranial synostosis OR unilateral coronal synostosis OR scaphocephaly OR trigonocephaly OR metopic synostosis OR sagittal synostosis OR lambdoid synostosis) AND (motor skills disorders OR developmental coordination disorders OR neurodevelopmental OR developmental disabilities OR developmental delay disorders)	100
Total		1.483

### Eligibility criteria

Studies were included if they met the following criteria: participants were children diagnosed with syndromic or non-syndromic CS; motor development was assessed using standardized motor development scales; quantitative data were reported; and the study design was cross-sectional, cohort, case–control or population based. Only studies assessing motor development in the preoperative period were considered eligible. Exclusion criteria comprised clinical trials; studies evaluating motor development exclusively in the postoperative period; case–control studies that did not report extractable data on the prevalence of motor delay among children with CS; studies in which it was not possible to calculate prevalence from the available data; abstracts, narrative or systematic reviews, case reports or case series, letters and editorials; studies without quantitative data; and studies with incomplete data for which no response was obtained from the authors. When multiple publications reported data from the same study population or cohort, only the study providing the most complete dataset relevant to the outcome of interest was included to avoid duplication of participants.

### Study selection

**Fig. 1:**
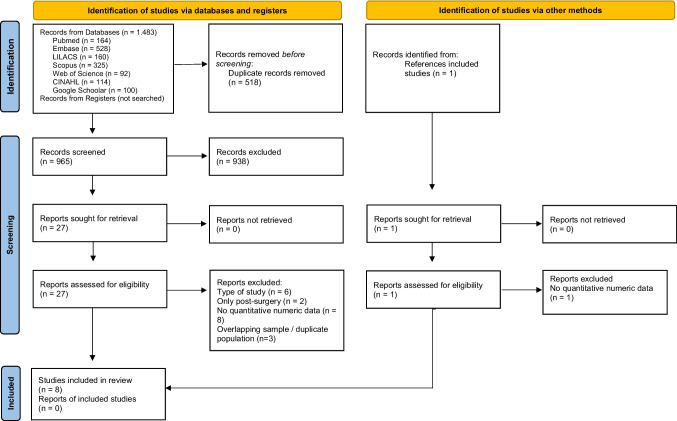
PRISMA 2020 flow diagram for new systematic reviews which included searches of databases, registers and other sources

Study selection was conducted in two phases. In Phase 1, all records identified across the databases were imported into Rayyan® (Qatar Computing Research Institute). Duplicate records were automatically identified and subsequently screened by one reviewer. Following a pilot test and reviewer training, titles and abstracts were screened to assess potential eligibility, and studies that clearly did not meet the predefined inclusion criteria were excluded at this stage. Discrepancies during Phase 1 were resolved through discussion between two reviewers, with the involvement of a third reviewer when necessary. In Phase 2, the full texts of the remaining studies were independently assessed by two reviewers to confirm eligibility according to the inclusion and exclusion criteria. The reasons for exclusion at the full-text stage were recorded (Fig. 1). Any disagreements were resolved through discussion and a final consensus meeting that included a third reviewer.

### Data extraction

Data extraction was performed independently by two reviewers, who thoroughly examined each included study. Extracted data were compared to minimize potential errors, and discrepancies were resolved by consensus or with the involvement of a third reviewer. The following information was extracted: author and year of publication, country of origin, study design, sample size, type of CS (syndromic or non-syndromic), motor assessment instrument, mean age of participants, main motor development outcomes, and quantitative results. When available, continuous data were extracted as means and standard deviations (SD). All efforts were made to ensure data completeness. Missing information was addressed by contacting corresponding authors via email. In the absence of a response within ten days, the study was excluded.

### Data synthesis and analysis

A prevalence meta-analysis was conducted in R (version 4.5.2) using a binomial–normal random-effects model based on Generalized Linear Mixed Models (GLMM) with a logit link function. Estimates were obtained on the logit scale and subsequently back transformed to proportions. Heterogeneity was assessed using Cochran’s Q, I^2^, and τ^2^, with τ^2^ estimated by maximum likelihood. Pooled prevalence estimates were reported with 95% confidence intervals (95% CI) and 95% prediction intervals, reflecting the expected variability in new populations. The use of GLMM was justified by its ability to preserve binomial variance and avoid continuity corrections, ensuring greater stability in studies with small sample sizes or extreme proportions. A significance level of 5% was adopted, and results were presented using forest plots.

### Risk of bias and methodological quality assessment

Methodological quality and risk of bias were independently assessed by two reviewers using the JBI Checklist for Prevalence Studies, which is appropriate for prevalence studies [25]. This tool evaluates domains related to potential sources of selection bias, measurement bias, confounding, data analysis, and reporting. Each item was rated as “yes,” “no,” or “unclear,” corresponding to low, high, or unclear risk of bias, respectively. Assessments were carefully reviewed by both reviewers, and discrepancies were resolved through discussion and consensus. When consensus could not be reached, a third reviewer was consulted. This approach ensured a rigorous and standardized appraisal of study quality, strengthening the reliability of the conclusions drawn from the synthesized evidence.

## Results

### Study selection and flow diagram

A total of 965 published studies were identified through database searches after the removal of duplicates. In addition, one article was identified from the reference list of a previous study [26]. During the second phase, 28 full-text articles were assessed for eligibility, of which 8 met the inclusion criteria. Appendix A (Table 3) presents the studies deemed ineligible and the respective reasons for exclusion. Accordingly, 8 articles published between 2001 and 2024 were included in the qualitative synthesis and analyzed in detail [13, 22, 23, 27, 28, 30, 31, 33]. Notably, only one of the included studies evaluated children with syndromic CS [23]. The study selection process is illustrated in the PRISMA flow diagram (Fig. 1).

### Characteristics of the included studies

The main characteristics of the samples from the 8 included studies are presented in Table 2. Most studies were conducted in the United States of America [27, 28, 30, 31, 33]. Overall, 625 pediatric participants (with or without motor delay) were assessed, including 510 children with CS and 115 controls. Regarding the assessment of motor development, 7 studies used the Bayley Scales of Infant Development (BSID), with four employing the BSID-II version [13, 27, 31, 33] and three using the BSID-III version [23, 28, 30]. Only one study employed the Standardized Neurodevelopmental Questionnaire (SNQ) [22].

**Table 2:** Summary of the key characteristics of the included studies

Study/Country/Local	Type of study	Total sample size (*n*)	Sample biological sex (*n*,%)	Age: range, (mean), SD	Type of CS (n, %) (suture involved)	Prevalence of CS in the total sample (*n*, %)	Method for motor assessment	Classification of the delay (*n*, %)	Prevalence of motor delay in CS (delayed)/sample = %
Cohen et al., 2004 United States of America Hospital	Prospective, multicenter study	22	F = 10, 45.5% M = 12, 54.5%	2.5–10 months (5.9), NI	Sagittal 10, 45.0% Metopic 5, 23.0% Unicoronal 7, 32.0%	22, 100%	BSDI-II	Mild Delay 5 (22.7%) Severely delayed 7 (31.8%) PDI: 79.5, SD = 19.65	(12)/22 = 54.2%
Da Costa et al., 2013 Australia Craniofacial clinic	Longitudinal	64	F = 18, 28.1% M = 46, 71.9%	4–32 months (21.2), 2.7	Sagittal 26, 40.6% Metopic 21, 32.8% Unicoronal 10, 15.6% Multisutural 7, 10.9%	64, 100%	BSDI-II	Mild Delay 12 (19.4%) Severely delayed 8 (11.3%) PDI: mean 87.8, SD = 12.5	(20)/64 = 30.7%
Imahiyerobo et al., 2019 United States of America Hospital	Prospective, transversal	77	F = 17, 22.1% M = 60, 77.9%	5.1 months (NI), 2.3	Sagittal 77, 100.0%	77, 100%	BSDI-III	NI	(14)/77 = 18.8%
Kunz et al., 2014 Germany University	Retrospective	25	F = 4, 16% M = 21, 84%	9.2 months (NI), NI	Metopic 25, 100.0%	25, 100%	SNQ	Mild Delay 6 (24%) Moderate Delay 4 (16%)	(10)/25 = 40%
Lynn et al., 2023 United States of America	Retrospective	66	F = 18, 27,3% M = 48, 72,7%	8.3 (3.4)	Sagittal 38 (57.6) Metopic 17 (25.8) Coronal 10 (15.1) Lambdoid 1 (1.5)	66, 100%	BSID-III	NI	(10)/66 = 15.2%
Panchal et al., 2001 United States of America Medical care center	Prospective	21	NI	NI (10.9), NI	Sagittal 11, 52.38 Metopic 5, 23.80 Bicoronal 2, 9.52 Unicoronal 3, 14.28	21, 100%	BSID-II	Mild delay 10 (47.6%) Severely delay 2 (9.5%) PDI: NI	(12)/21 = 57%
Ripstein et al., 2024 North America Medical Clinic	Retrospective cohort study	59 non-syndromic 8 syndromic	F = 22, 32.8% M = 45, 67.2%	7 months (5.25—10.7)	Sagittal 29, 43.3% Metopic 21, 31.3% Unicoronal 8, 11.9% Bicoronal 2, 3.0% Multisutural 6, 9.0% Unilambdoidal 1, 1.5%	67, 100%	BSID III	Global Developmental Delay non syndromic 6 (8.9%) Global Developmental Delay syndromic 6 (8.9%)	(12)/67 = 17.9%
Starr et al., 2007 United States of America Medical care center/Hospital	Prospective multicenter longitudinal study with a control group	283 CRSG: 168 CG: 115	CRSG: F = 61, 36% M = 107, 64% CG: F = 47, 41% M = 68, 59%	NI	Sagittal 86, 51.19% Metopic 35, 20.83% Unicoronal left 16, 9.52% Unicoronal right 20, 11.90% Lambdoid 11, 6.54%	168, 67.2%	BSID-II	NI	CRSG: (77)/168 = 45% CG: (50)/168 = 30%

*BSID-II* bayley scales of infant development-2, *BSID-III* bayley scales of infant development-3, *CS* craniosynostosis, *CRSG* craniosynostoses group, *CG* control group, *F* female, *M* male, *n* number, *NI* not informed, *PDI* psychomotor developmental index, *SNQ* standardized neurodevelopmental questionnaire

Regarding genetic characterization, the rigor of screening protocols varied across the literature. Only Starr et al. (2007) [27] reported systematic gene sequencing to exclude syndromic cases, while Kunz et al. (2014) [13] performed genetic consultations based on clinical suspicion. Ripstein et al. (2024) [23] explicitly included a subgroup of eight children (11.9%) with syndromic CS. The remaining five studies classified their samples as non-syndromic based primarily on clinical dysmorphology, without reporting standardized molecular protocols or specific genetic testing.

### Results of individual studies

#### Assessment of motor development using the BSID-II

Cohen et al. (2004) [27] compared pre- and postoperative neurodevelopment in 22 children aged 2.5 to 10 months diagnosed with non-syndromic CS. Development was assessed at two time points—two months before surgery and one year after the procedure. For the purposes of this review, only preoperative data referring to the Psychomotor Development Index (PDI) were considered. The mean preoperative PDI score was 79.5 (SD = 19.65; range: 50–117), which was significantly lower than expected (*p* < 0.0001). Among participants, seven showed significant delay, five mild delay, nine were within normal limits, and only one demonstrated accelerated performance. When results were analyzed according to the affected suture, no statistically significant differences were observed between the coronal (mean = 72.9) and sagittal (mean = 89.2) groups.

Da Costa et al. (2013) [13] aimed to assess the prevalence of cognitive and motor deficits before and/or after CS repair, as well as the risk of delay according to the affected suture, in a sample of 63 infants. The mean PDI was significantly lower than population norms (t (1,62) = 7.80; *p* < 0.001), falling within the range of mild delay (95% CI: 84.5–90.8). Risk ratio analyses indicated that children with CS were 2.5 times more likely to perform below average compared with the normative population. The proportion of children with mild or severe delay (31.7%) exceeded that expected in the general population (14.8%). No participants demonstrated accelerated performance.

Panchal et al. (2001) [31] evaluated 21 children with non-syndromic CS prior to surgical intervention and compared them with normative data. The distribution of PDI scores differed significantly from the reference population (*p* < 0.001). No participants demonstrated accelerated performance; 43% had normal scores, 48% exhibited mild delay, and 9% showed significant delay.

Starr et al. (2007) [33] compared pre- and postoperative development in 168 children with CS and 115 unaffected controls. PDI scores indicated significantly poorer motor performance in the CS group (mean = 84.85) compared with controls (mean = 88.93), with a mean difference of –4.04 points (95% CI: –6.78 to –1.29; *p* = 0.004).

#### Assessment of motor development using the BSID-III

Imahiyerobo et al. (2019) [28] examined risk factors for developmental delay in 77 patients with sagittal CS who had not yet undergone surgery. Motor development was assessed using the BSID-III in children aged 2 to 12 months. Fourteen infants (18.2%) scored at or below the ninth percentile and were classified as having motor delay.

Lynn et al. (2023) [30] conducted a longitudinal (pre- and postoperative) evaluation of neurodevelopment in 66 patients with non-syndromic CS, also using the BSID-III. The mean preoperative motor composite score (95.2 ± 13.7) was significantly lower than the normative mean (*p* = 0.005), with 15.2% of children presenting motor delay—a proportion similar to that reported by Imahiyerobo et al. (2019) [28].

Complementing these findings, Ripstein et al. (2024) [23] investigated 67 preschool-aged children awaiting surgery, comprising 59 non-syndromic and eight syndromic CS cases. Overall, 12 children (17.9%) exhibited motor delay; notably, this impairment was equally distributed in absolute numbers between the two groups (six cases each). However, this represents a disproportionately higher prevalence within the syndromic subgroup (75% vs. 10.2%), underscoring the significant developmental burden associated with syndromic CS.

#### Assessment of motor development using the SNQ

Among studies employing instruments other than the BSID, only one used the SNQ. Kunz et al. (2014) [22] evaluated 25 children with metopic CS using the Standardized Neurodevelopmental Questionnaire (SNQ), which assesses gross motor function, manual coordination, speech, and cognition. Deficits were classified as mild, moderate, or severe based on delays relative to typical developmental milestones. Twelve children (48%) demonstrated typical development, whereas 10 (40%) exhibited some degree of deficit—predominantly mild (*n* = 6) or moderate (*n* = 4). As this was the only study employing an instrument different from those used in the remaining studies, the findings of Kunz et al. (2014) [22] complement but cannot be directly compared with estimates derived from studies using the BSID-II and BSID-III.

#### Quantitative synthesis of results

**Fig. 2:**
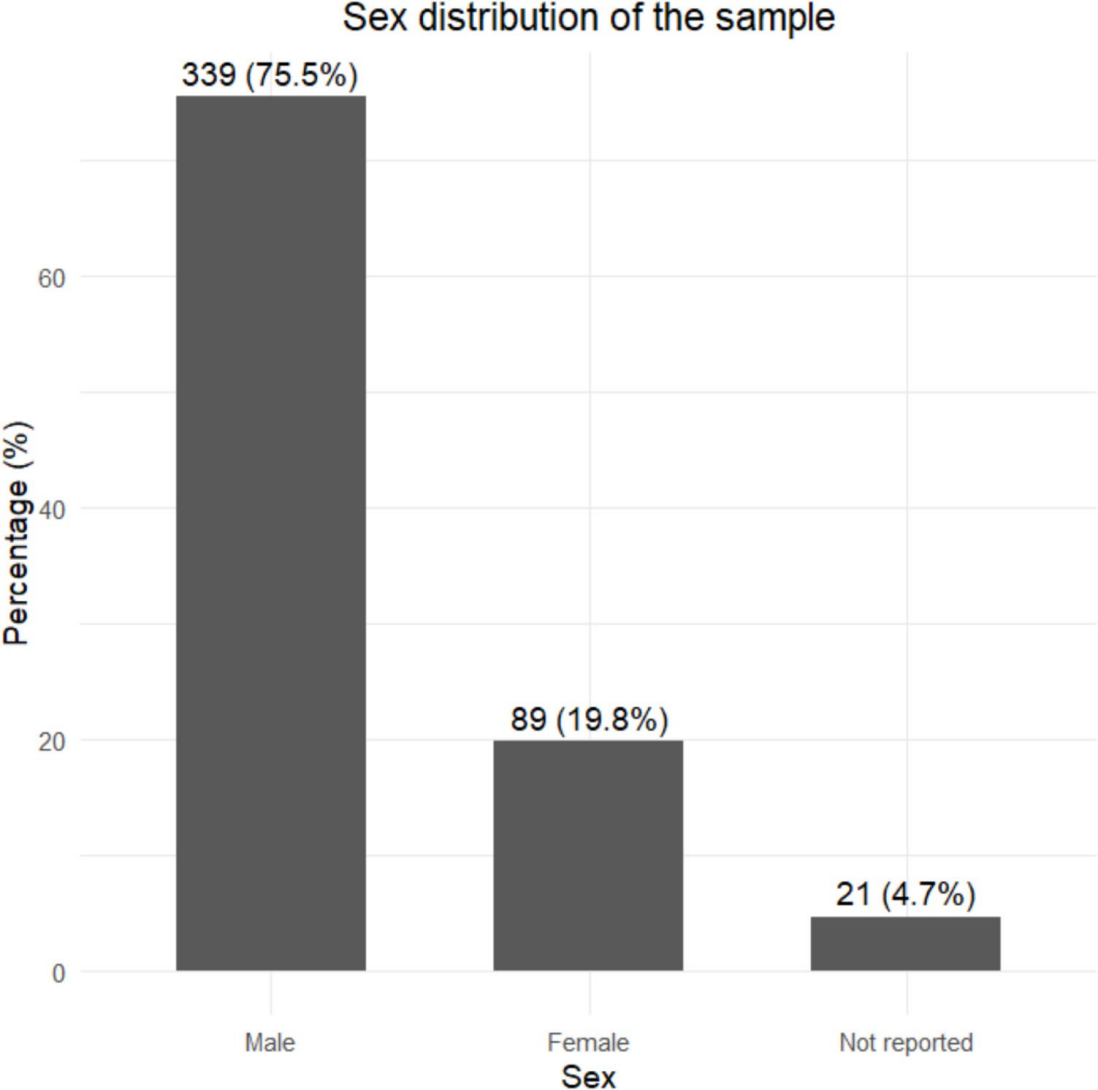
Sex distribution of participants across included studies

**Fig. 3:**
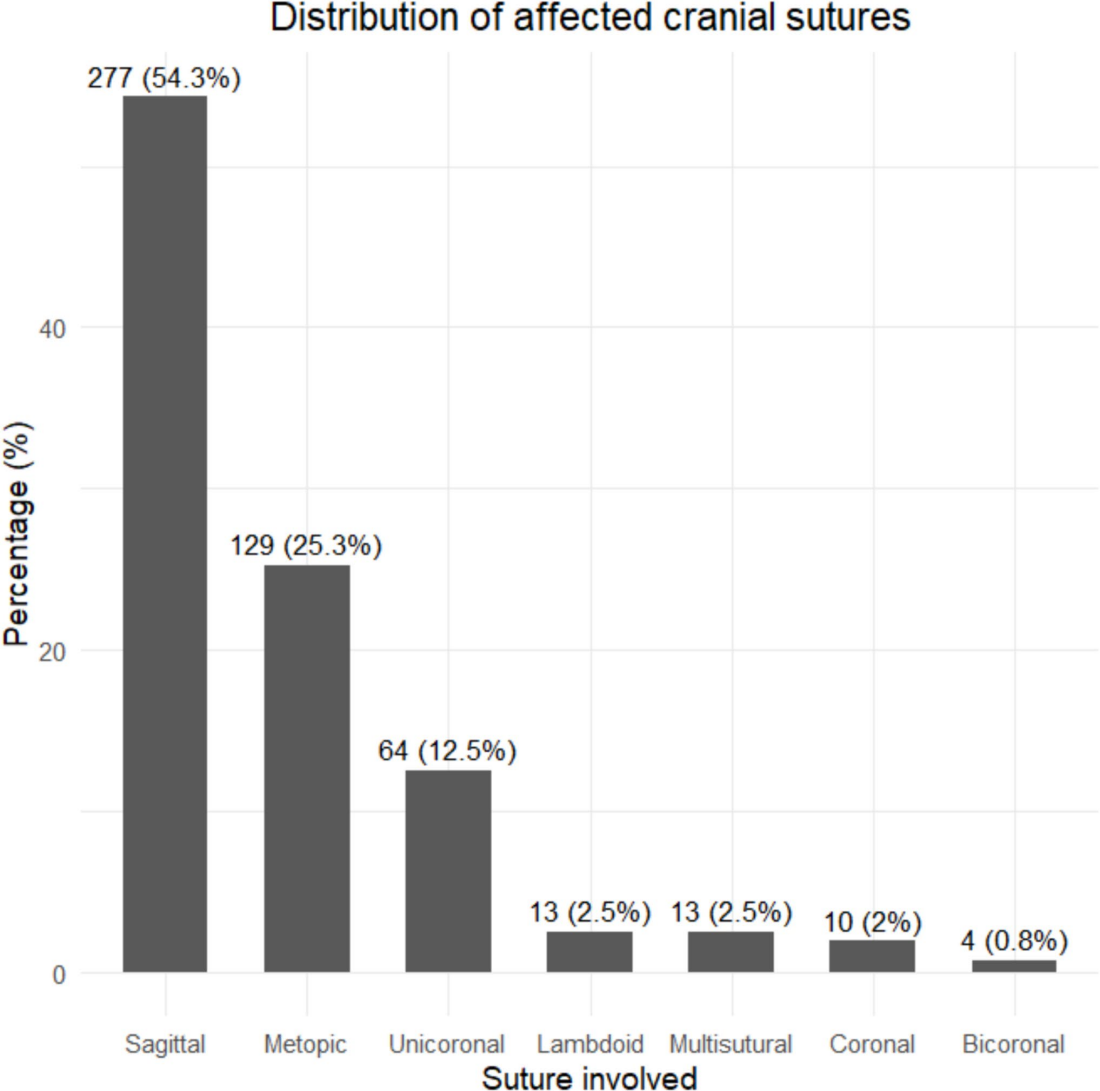
Distribution of affected suture types among patients with CS

Regarding participant characteristics, children ranged in age from 2 to 32 months, with a predominance of males 339 boys and 89 girls); only one study did not report participant sex [31]. In terms of clinical characteristics, sagittal suture involvement was the most frequent, accounting for 54,3% of cases. These data are detailed in Figs. 2 and 3.

#### Prevalence of motor delay in children with CS

**Fig. 4:**
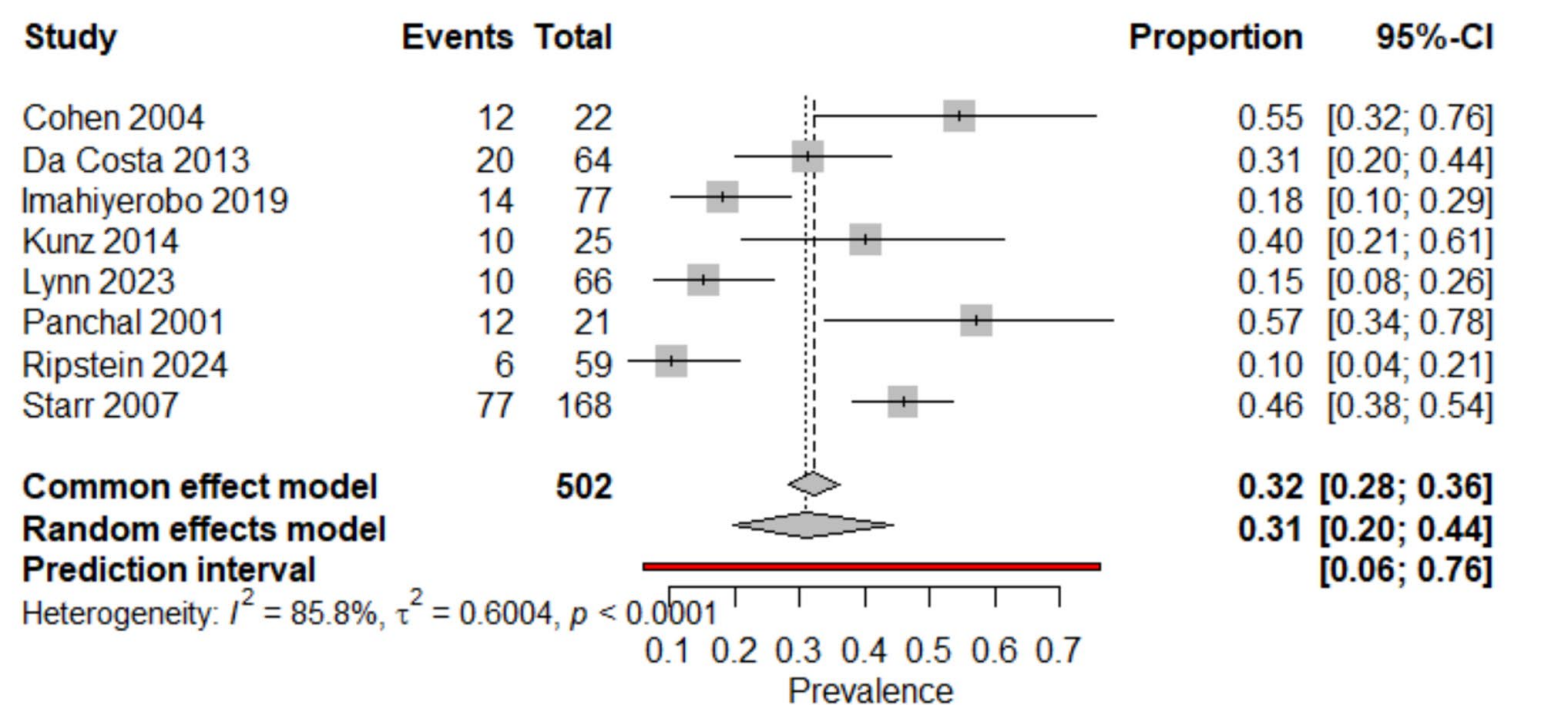
Forest plot of motor delay prevalence in children with CS

A total of 8 studies (*n* = 510 children with CS) were included, of which 167 presented with motor delay. The prevalence meta-analysis yielded a pooled prevalence of 0.31 (95% CI: 0.20–0.44). Substantial heterogeneity was observed (I^2^ = 85.8%, τ^2^ = 0.6004, Cochran’s Q test *p* < 0.0001). The 95% prediction interval ranged from 0.06 to 0.76, indicating wide variability. For reference, the common-effect model estimated a prevalence of 0.32 (95% CI: 0.28–0.36). Due to the small number of syndromic cases identified (*n* = 8) compared to the non-syndromic cohort (*n* = 502), a formal subgroup comparison was not statistically feasible. These findings support the premise that children with CS are at increased risk of motor delay, although the magnitude of this risk varies according to sample characteristics and methodological features of the included studies (Fig. 4).

#### Risk of bias and methodological quality

**Fig. 5:**
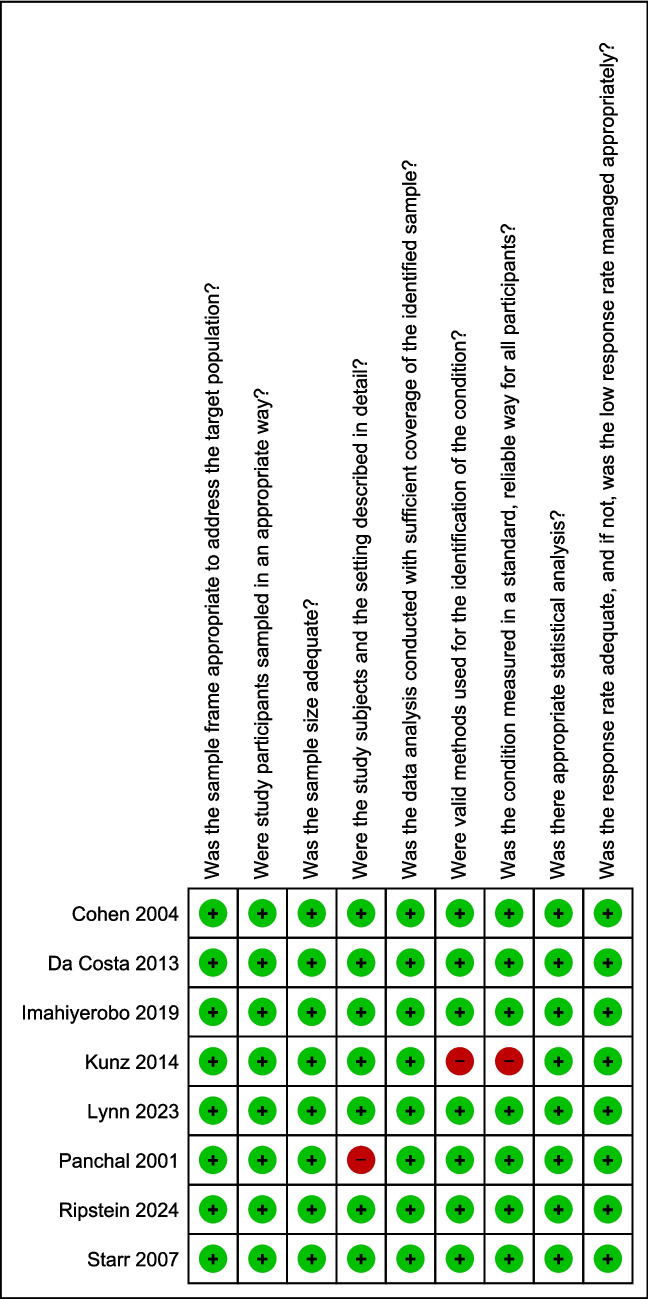
Risk of bias summary according to the JBI Critical Appraisal Checklist for Prevalence Studies: review authors’ judgments about each risk of bias item for each included study

**Fig. 6:**
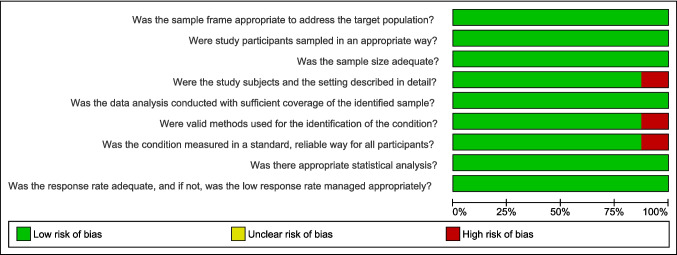
Risk of bias graph according to the JBI Critical Appraisal Checklist for Prevalence Studies: review authors’ judgments about each risk of bias item presented as percentages across all included studies

Methodological assessment of the included studies indicated that most exhibited a low risk of bias, with maximum scores (9/9) assigned to Cohen et al. (2004) [27]; Da Costa et al. (2013) [13]; Imahiyerobo et al. (2019) [28]; Lynn et al. (2023) [30]; Ripstein et al. (2024) [23]; and Starr et al. (2007) [33]. These studies demonstrated adequate sample selection, clear descriptions of participants and settings, valid and standardized methods for condition identification, and appropriate statistical analyses (Fig. 5 and 6).

Panchal et al. (2001) [31] received a score of 8/9, primarily due to the absence of sex distribution reporting, which limited participant characterization and the exploration of potential sex-related differences. Kunz et al. (2014) [22] achieved a score of 7/9, owing to the use of a non-validated questionnaire and limited replicability, which may have reduced the precision of prevalence estimates.

Despite these isolated limitations, the overall methodological quality of the included studies was considered satisfactory, supporting the reliability of the prevalence estimates of motor delay in children with CS presented in this review.

## Discussion

This systematic review and meta-analysis synthesized data from 8 studies comprising 502 children with non-syndromic CS and demonstrated that approximately one third of these children (31%; 95% CI: 20–44%) exhibited motor delay already in the preoperative period. Overall, these findings indicate that roughly one in three children present with some degree of motor impairment, highlighting the frequency of motor involvement in this population. Derived from a quantitative synthesis of the available studies, these results consistently show that motor impairment is a frequent manifestation of CS, regardless of the affected suture subtype or the assessment instrument used. Although the magnitude of the effect varied across studies, the convergence of findings points to a clear pattern of motor vulnerability in this population, underscoring the importance of early screening and standardized motor development assessment in clinical practice.

Comparison with previous literature reinforces these results. Observational studies have suggested that children with CS are at increased risk of motor and cognitive developmental delays, which may persist even after surgical correction [13, 14, 33]. These findings support the premise that children with CS are at elevated risk of motor delay, although the magnitude of this risk varies according to sample characteristics and methodological features of the studies.

The substantial heterogeneity observed in the meta-analysis (I^2^ = 85.8%) indicates the presence of marked methodological differences among the included studies. A central source of this variability was the use of different instruments to assess motor development—namely the BSID-II, BSID-III, and SNQ— which differ in structure, scoring systems, and normative samples. Importantly, studies using the BSID-II were employing the version that was appropriate at the time of data collection, since the BSID-III was only published in 2006 [34, 35]. Previous research has suggested that the two versions of the Bayley scales may yield different score distributions, with some evidence indicating lower motor scores with the BSID-II in typically developing infants and higher scores with the BSID-III in populations at developmental risk [37, 38]. These differences highlight the importance of interpreting results in relation to contemporaneous control groups assessed with the same instrument, rather than relying solely on normative comparisons. In addition, consistent reporting of score distributions (e.g., typical development, mild delay, severe delay) and clearly defined cutoff criteria would facilitate more reliable comparisons across studies.

In addition, one study (Imahiyerobo et al., 2019) applied a slightly more conservative threshold to define motor delay, corresponding to approximately 1.5 SD below the mean, whereas most studies defined mild delay as 1 SD below the mean. Because the present meta-analysis aimed to estimate the prevalence of motor delay (presence vs. absence of delay) rather than the severity of impairment, this study was retained in the pooled analysis. Nevertheless, differences in cutoff thresholds may influence prevalence estimates and should be considered when interpreting the pooled results, as they may also contribute to between-study heterogeneity.

Age at assessment constituted another relevant source of heterogeneity. Motor maturation during the first two years of life is rapid and non-linear, and small variations in age (chronological or corrected) can substantially influence standardized test scores [39]. During this period, interindividual variability is greater and classification stability is lower, which helps explain part of the divergence across studies. In the studies included in this review, a considerable proportion of samples consisted of very young infants, particularly those assessed before six months of age developmental window in which transient delays are common and motor maturation is still highly variable. In contrast, investigations assessing older children tended to identify more stable and consolidated deficits [40, 41]. As most studies did not stratify results by age groups, it was not possible to precisely determine the impact of age distribution on pooled estimates; nevertheless, the predominance of younger infants may have contributed to the observed variability. Future studies that structure analyses by age strata—especially distinguishing children younger and older than six months—may provide more precise estimates and reduce part of the heterogeneity detected in this synthesis.

Differences among CS subtypes also contributed to variability in the findings, in a manner consistent with the prevalence pattern observed in the included studies. Sagittal synostosis, which accounted for the majority of cases in the present review (57%), has been associated in neuroimaging studies with alterations in functional connectivity within frontoparietal and prefrontal circuit regions implicated in motor planning, visuospatial integration, and executive functions [42, 43]. Metopic synostosis, although less frequent in the analyzed studies, exhibits relevant anatomical particularities; evidence indicates reduced global white matter volume and an anomalous growth pattern, with relative increases in gray matter in the cingulate and temporal lobes, as well as altered frontal white matter microstructure on diffusion imaging [44, 45]. Unicoronal synostosis, also less prevalent, has been associated with atypical connectivity patterns that may affect networks involved in bilateral motor organization and visuospatial processing. A recent neuroimaging review by Russo et al. (2024) [46] reinforced that each suture produces a distinct pattern of cranial deformation, with variable impact on frontal, parietal, and orbital lobes. Thus, the anatomical and functional specificities of each subtype may help explain, at least in part, the variability observed across studies investigating motor performance in CS.

From a pathophysiological perspective, multiple pathways may contribute to the observed motor delay. Premature suture fusion restricts the space available for brain growth and may, in some cases, lead to increased intracranial pressure (ICP), cortical tissue deformation, and alterations in cerebral architecture [47]. Neuroimaging studies in children with CS demonstrate that frontoparietal and cerebellar regions—critical for motor control, balance, and sensorimotor integration—may exhibit atypical configurations, with differences in volume, cortical thickness, and gyral organization compared with typically developing children [48]. Although elevated ICP is not present in all patients, craniocerebral imbalance may lead to neuro-ophthalmological alterations, corneal deformation due to incomplete eyelid closure, strabismus, or refractive disorders [49, 50]. In this context, impairments in visual sensory input, spatial perception, and gaze stability may negatively affect tasks requiring fine postural control, visuomotor coordination, and manual dexterity, thereby contributing to delayed acquisition of motor milestones [48, 51].

Beyond macroscopic alterations, evidence also points to microstructural and connectivity changes in white matter tracts essential for motor coordination, such as the corpus callosum, superior longitudinal fasciculus, and thalamic radiations [52]. Persistent differences in frontocerebellar and interhemispheric connectivity have been described in older children, around 12 years of age, even after surgical correction, suggesting that the effects of CS on brain development may be long-lasting [42, 43]. Collectively, these findings support a model in which mechanical cranial constraints, anatomical remodeling, and altered maturation of white matter networks interact to produce an atypical developmental trajectory, in which the acquisition of gross and fine motor skills may occur more slowly or less efficiently [52, 53].

This trajectory may be further modulated by underlying genetic factors. Recent advances have identified pathogenic variants in genes such as SMAD6 (SMAD family member 6) and ERF (ETS2 Repressor Factor) in patients previously categorized as having isolated CS, highlighting that these genotypes show variable penetrance and can substantially influence neurodevelopmental phenotypes [54, 55]. Heterozygous loss-of-function variants in ERF and rare damaging variants in SMAD6 increase the risk of CS and are associated with a spectrum of neurodevelopmental outcomes, including language and motor delay, learning difficulties and behavioral problems, even in children without a classic syndromic phenotype [56, 57].

In summary, this review demonstrates that children with non-syndromic CS exhibit a substantial prevalence of motor delay risk. Although only one included study performed a direct comparison with a control group, its findings reinforce this trend: 45% of children with CS (77/168) exhibited motor delay, compared with 30% (50/168) in the control group [33]. This result converges with the recent consensus of the Craniosynostosis Research Network reported by Baraya et al. (2025) [58], which indicates poorer motor performance and a two- to threefold increased risk of delay, particularly in cases involving multiple sutures.

However, the distribution of CS subtypes among the included studies largely reflects their epidemiological prevalence, with sagittal synostosis being the most frequent subtype. Therefore, the available evidence does not allow a direct inference regarding an increased risk of motor delay associated with a specific suture type. The findings of this meta-analysis suggest that the risk of motor delay in children with CS may be influenced by multiple factors, including anatomical characteristics, neurobiological mechanisms related to brain growth and cortical organization, and methodological aspects such as the assessment instruments used, age at evaluation, and cutoff criteria. These results reinforce the need for systematic motor development screening and longitudinal follow-up throughout childhood, in line with consensus recommendations that emphasize structured neurodevelopmental monitoring protocols integrating motor, language, cognitive, and behavioral assessments. Thus, the findings of the present meta-analysis provide quantitative evidence that supports and strengthens these recommendations.

Nevertheless, several limitations should be acknowledged. First, potentially relevant studies were excluded because they did not specify, in numerical terms, the number of children with motor delay or failed to provide normative data that would allow prevalence recording. In practice, many studies merely report the presence of delay without detailing the proportion of affected children, which restricts their inclusion in quantitative analyses. Additionally, several studies did not consistently report important sample parameters, such as mean age, age distribution, and sex, which are potential moderators and essential for result interpretation.

Furthermore, a significant limitation across the included studies is the lack of standardized genetic testing and detailed reporting of molecular data. Most studies classified patients as ‘non-syndromic’ based primarily on clinical dysmorphology rather than comprehensive genomic screening. The absence of systematic screening for these variants in the reviewed literature introduces a potential ascertainment bias, as the observed motor delays might be partially attributed to unrecognized genetic predispositions rather than the mechanical effects of the synostosis alone.

Standardization of methodological reporting, stratification by suture subtype, incorporation of neuroimaging techniques into longitudinal designs and and the inclusion of comprehensive genetic panels may, in future investigations, more precisely elucidate the relationships between genotype, cranial remodeling, brain development, and motor performance in children with CS syndromic or non-syndromic. Future studies should systematically report the number and proportion of children classified as delayed according to predefined criteria, thereby enabling comparability across studies.

Despite these limitations, no consolidated quantitative estimate of motor delay prevalence encompassing all affected sutures had previously been available. By synthesizing data from eleven studies, this review provides a novel and comprehensive estimate, enabling not only quantification of prevalence but also exploration of clinical and methodological factors contributing to variability across studies.

## Conclusion

This systematic review and meta-analysis demonstrate that approximately one in three children with non-syndromic CS (31%), aged 2 to 32 months and predominantly male, and affected by sagittal, metopic, unicoronal, lambdoid, and multisuture subtypes, present with motor delay. Despite heterogeneity across studies, premature fusion of the cranial sutures is consistently associated with increased vulnerability in motor development, regardless of the affected suture subtype or the assessment instrument used.

These findings reinforce the need for standardized preoperative motor development screening in children with CS. Furthermore, future research should prioritize the standardization of assessment scales, stratification by affected suture subtype, and precise reporting of age and timing of assessment to improve comparability across studies and support more robust evidence synthesis.

## Data Availability

No datasets were generated or analysed during the current study.

## Appendix A

**Table 3:** List of excluded studies and reasons for exclusion (*n* = 20)

Author/Year	Excluded (reason)
Bellew et al., 2005 [59]	Lack of quantitative data
Bellew et al., 2019 [60]	Lack of quantitative data
Chieffo et al., 2016 [61]	Abstract only
Da costa et al., 2012 [36]	Overlapping sample/duplicate population
Fontana et al., 2018 [62]	Lack of quantitative data
Gray et al., 2014 [63]	Lack of quantitative data
Johns et al., 2017 [64]	Abstract only
Johns et al., 2022 [65]	Abstract only
Kapp-Simon et al., 2005 [29]	Overlapping sample/duplicate population
Knight et al., 2012 [66]	Abstract only
Larysz D., 2012 [67]	Abstract only
Moreno-Villagómez et al., 2021 [40]	Postoperative assessment only
Moreno-Villagómez et al., 2014 [68]	Postoperative assessment only
Naran et al., 2016 [69]	Abstract only
Ruiz-Correa et al., 2007 [32]	Overlapping sample/duplicate population
Shimoji et al., 2015 [70]	Lack of quantitative data
Starr et al., 2010 [71]	Lack of quantitative data
Star et al., 2012 [72]	Lack of quantitative data
Speltz et al., 1997 [73]	Lack of quantitative data
Speltz et al., 2007 [26]	Lack of quantitative data
